# Potentially Inappropriate End of Life Care and Healthcare Costs in the Last 30 Days of Life in Regions Providing Integrated Palliative Care in the Netherlands: A Registration-based Study

**DOI:** 10.5334/ijic.7504

**Published:** 2024-07-08

**Authors:** Chantal F. R. Pereira, Anne-floor Q. Dijxhoorn, Berdine Koekoek, Monique van den Broek, Karin van der Steen, Marijanne Engel, Marjon van Rijn, Judith M. Meijers, Jeroen Hasselaar, Agnes van der Heide, Bregje D. Onwuteaka-Philipsen, Marieke H. J. van den Beuken-van Everdingen, Yvette M. van der Linden, Manon S. Boddaert, Patrick P. T. Jeurissen, Matthias A. W. Merkx, Natasja J. H. Raijmakers

**Affiliations:** 1 Netherlands Comprehensive Cancer Organisation (IKNL), Utrecht, The Netherlands; 2 Netherlands Association for Palliative Care, Utrecht, The Netherlands; 3 Center of Expertise in Palliative Care, Leiden University Medical Center, Leiden, The Netherlands; 4 Gelre Hospitals, Apeldoorn, Netherlands Apeldoorn, The Netherlands; 5 Synchroon, Oss, The Netherlands; 6 Proscoop, Zwolle, The Netherlands; 7 Center of Expertise in Palliative Care Utrecht, Julius Center for Health Sciences and Primary Care, University Medical Center Utrecht, Utrecht, the Netherlands; 8 Department of Medicine for Older People, Amsterdam Public Health Research Institute, Amsterdam UMC –Vrije Universiteit Amsterdam, Amsterdam, The Netherlands; 9 Center of Expertise Urban Vitality, Faculty of Health, Amsterdam University of Applied Science, Amsterdam, the Netherlands; 10 Department of Health Services Research, CAPHRI School for Public Health and Primary Care, Faculty of Health Medicine and Life Sciences, Maastricht University, Maastricht, The Netherlands; 11 Zuyderland Care, Zuyderland Medical Center, Sittard-Geleen, The Netherlands; 12 Department of Anaesthesiology, Pain and Palliative Medicine, Radboud University Medical Center, Nijmegen, The Netherlands; 13 Department of Public Health, Erasmus MC, University Medical Center Rotterdam, Rotterdam, The Netherlands; 14 Department of Public and Occupational Health, Amsterdam Public Health Research Institute, Amsterdam UMC –. Vrije Universiteit Amsterdam, Amsterdam, The Netherlands; 15 Center of Expertise in Palliative Care, Maastricht University Medical Center (MUMC+), Maastricht, The Netherlands; 16 Radboud Institute for Health Sciences, Scientific Center for Quality of Healthcare (IQ healthcare), Radboud University Medical Center, Nijmegen, The Netherlands

**Keywords:** palliative care, integrated palliative care, potentially inappropriate end of life care, healthcare costs, Medicine, Nursing, Health economics, Deceased adults

## Abstract

**Introduction:**

This study aimed to assess the effect of integrated palliative care (IPC) on potentially inappropriate end- of-life care and healthcare-costs in the last 30 days of life in the Netherlands.

**Methods:**

Nationwide health-insurance claims data were used to assess potentially inappropriate end-of-life care (≥2 emergency room visits; ≥2 hospital admissions; >14 days hospitalization; chemotherapy; ICU admission; hospital death) and healthcare-costs in all deceased adults in IPC regions pre- and post- implementation and in those receiving IPC compared to a 1:2 matched control group.

**Results:**

In regions providing IPC deceased adults (n = 37,468) received significantly less potentially inappropriate end-of-life care post-implementation compared to pre-implementation (26.5% vs 27.9%; p < 0.05). Deceased adults who received IPC (n = 210) also received significantly less potentially inappropriate end-of-life care compared to a matched control group (14.8% vs 28.3%; p < 0.05). Mean hospital costs significantly decreased for deceased adults who received IPC (€2,817), while mean costs increased for general practitioner services (€311) and home care (€1,632).

**Discussion:**

These results highlight the importance of implementation of integrated palliative care and suitable payment. Further research in a larger sample is needed.

**Conclusion:**

This study shows less potentially inappropriate end-of-life care and a shift in healthcare costs from hospital to general practitioner and home care with IPC.

## Introduction

Owing to population aging and the expected increase in non-communicable diseases, the number of people facing life-threatening illnesses or frailty, and therefore in need of palliative care, will substantially increase over the coming years [1]. The World Health Organization (WHO) defines palliative care as an approach that improves the quality of life of patients and their families facing the problems associated with life-threatening illness, through the prevention and relief of suffering by means of early identification and impeccable assessment and treatment of pain and other problems; physical, psychosocial, and spiritual [2]. Palliative care extends from the diagnosis of life-threatening illnesses or frailty to the patient’s end of life [3,4]. Research has shown that palliative care improves patients’ quality of life and care [5,6,7] and is associated with lower health care expenditures [8,9,10,11] and less potentially inappropriate end-of-life care [12,13,14].

Potentially inappropriate end-of-life care consists of treatments and care in the last months of a patient’s life for which the expected health benefits do not outweigh the expected adverse outcomes [15]. For example, although admission to an intensive care unit (ICU) near death may be beneficial in individual cases, it has been shown to be an aggregated determinant of poor quality of life at the end of life [16]. Therefore, potentially inappropriate end-of-life care cannot be used to assess the quality of care at the individual level but can be used as a population-based indicator of the performance of a healthcare system with regard to end-of-life care. In addition, potentially inappropriate end-of-life care may be considered an economic waste within the healthcare system because the resources spent on it cannot be used for other, more beneficial purposes. In the United States, it has been estimated that 44.1 billion dollars spent annually on end-of-life care can be considered an economic waste [17]. Nevertheless, previous studies have reported substantial percentages of patients receiving potentially inappropriate end-of-life care. In high-income countries, visits to the emergency room (ER) range from 25.0% to 68.0%, and in-hospital deaths range from 25.1% to 74.8% [18,19,20], indicating the need for improvement in end of life care.

As patients in a palliative care trajectory often receive care from multiple healthcare professionals in various settings [21], one of the impeding factors in improving quality of care at the end of life is adequate coordination and continuity of care [22,23]. Patients who receive palliative care report greater satisfaction with care [24], but continuity is a challenge due to fragmentation in the healthcare system [22]. Overcoming this challenge requires a patient-centered approach that integrates all services involved throughout the palliative care trajectory [25,26]. In other areas of health care, such as Parkinson’s disease care and maternity care, integrated care is being used to achieve this [27,28]. The WHO defines integrated care as care that aims to enhance quality of care and quality of life, consumer satisfaction, and system efficiency [29]. In the Netherlands, integrated palliative care initiatives such as case management, palliative care pathways, and multidisciplinary specialist palliative care teams are on the rise [30,31,32].

Studies on potentially inappropriate end-of-life care and healthcare costs in the last phase of life are currently limited to palliative care interventions provided within a single care setting [9,14]; furthermore, evidence on the effects of integrated palliative care is lacking. Therefore, this study aims to assess the effect of integrated palliative care on potentially inappropriate end-of-life care and healthcare costs in the last 30 days of life of deceased patients in different regions of the Netherlands.

## Methods

### Study design

This observational study used nationwide health insurance claims data to assess potentially inappropriate end-of-life care and healthcare costs in the last 30 days of life for all deceased adults in five integrated palliative care initiatives, before and after the implementation of integrated palliative care. In addition, deceased adults who received integrated palliative care from one of these initiatives were compared with a 1:2 matched control group. This study was exempt from medical ethical review according to the Dutch Medical Research Involving Human Subjects Act (WMO) and was approved by the Medical Research Ethics Committee of Brabant (number NW2021-41).

### Setting and study population

The study population was selected from an inventory within all regions of the Netherlands [30]. In this inventory, all integrated palliative care initiatives in the Netherlands were invited to share documentation of their interventions. The quality of all initiatives was evaluated by two independent reviewers based on the key elements derived from the Netherlands Quality framework for Palliative Care. [22]. Based on the documentation shared by the initiatives, the researchers scored the level of implementation of each key element. Highest possible score was 23 (Supplementary File 1). Subsequently, a consensus on evaluation was reached through a discussion between the two reviewers, led by an independent researcher. A total of 39 initiatives were evaluated. 14 initiatives had a score of ≥10. Of these, six initiatives were selected based on geographic distribution, the implementation of multiple key elements, variation in the intervention, and hospital involvement [30]. One of these six was excluded because the implementation of integrated palliative care in this initiative was delayed until after the study period. The remaining five initiatives were included in the study. Subsequently, all deceased adults (including those who died unexpectedly) within the regions of these five integrated palliative care initiatives between the period 2015–2019 were included in the analysis.

As they were selected from existing initiatives, each initiative had started implementation at different times. Therefore, the period before the implementation of each initiative is defined as the pre-implementation period for this analysis. The three months after start of the implementation of each initiative was included in the pre-implementation period, as the healthcare professionals involved needed this time to adjust care provision from standard care to the intervention. The period thereafter was defined as the post-implementation period for this analysis. The duration of the post-implementation period varied among the initiatives from 0.25 to 3.75 years (Supplementary File 2).

### Intervention: Integrated palliative care initiatives

Although the WHO has offered a definition of integrated care, it has also stated that it is difficult to achieve a unifying understanding of the concept, as the different stakeholders involved have different views and expectations thereof [29]. Independent of the perspectives of the stakeholders involved, integrated care requires integration at different clinical and organizational levels [33]. This is in line with the Netherlands Quality framework for Palliative Care, according to which palliative care “requires an interdisciplinary approach and calls for a good collaborative relationship among disciplines” [p.30] [34]. To facilitate this collaboration, “the organizations involved in a region work together effectively and efficiently” [p.37]. In addition to the integration of different levels of healthcare, generalist palliative care in the Netherlands is provided in different care settings by all healthcare professionals involved with patients facing life-threatening illnesses or frailty. In complex cases, healthcare professionals with extra training and experience in palliative care can be asked to provide specialised palliative care [35]. Integrated palliative care initiatives in the Netherlands, therefore, aim to integrate healthcare from different care settings as well as generalist and specialist palliative care; in standard care, different healthcare professionals involved with the patient in different care settings often provide palliative care independently, and the involvement of specialist palliative care is suboptimal [36].

Care pathways, case management, and multidisciplinary teams have been identified as potential interventions to achieve integrated care by the WHO [37]. The five selected initiatives used one or more of the elements to achieve integrated palliative care: 1) integrated specialist palliative care case management, 2) integrated palliative care pathways, and 3) integrated specialist palliative care teams (Supplementary File 2). Integrated case management entails the assignment of a nurse specialized in palliative care to patients with palliative care needs. The nurse supports patients, relatives, and all involved healthcare professionals throughout the palliative care trajectory. Integrated care pathways describe all steps in the care pathway, including the responsibilities of the generalist and specialist palliative care healthcare professionals involved in all settings. The integrated specialist palliative care teams are multidisciplinary and comprise healthcare professionals specialized in palliative care and working in primary, secondary, and/or tertiary care settings. The team can be consulted by generalists in palliative care across all settings. Services by the team included consultations by generalist palliative care professionals, case discussions in multidisciplinary team meetings (MDTs), and provision of specialized palliative patient care.

### Data collection

Data were obtained from Vektis B.V, a nationwide health insurance claims database in the Netherlands. Because it is legally mandatory for all people living or working in the Netherlands to have health insurance, this database covers almost all residents of the country. Vektis uses encrypted health card numbers to collect patient-level insurance data from primary, secondary, and tertiary care settings. The initiatives in this study recorded patients who received integrated palliative care. Patients were identified in the Vektis database using probabilistic matching based on their date of birth, gender, and date of death.

### Outcomes

Sociodemographic characteristics included age (in 10-year categories), gender, cancer diagnosis (yes or no), year of death, and region. Cancer diagnosis was determined by the presence of a diagnosis-treatment combination (DTC) for solid tumours in the year preceding death (Supplementary File 3) [14]. Hospital care is reimbursed for DTC in the Netherlands. A DTC includes all hospital services related to diagnosis, treatment, and follow-up per patient. To assess the quality of care, six population-based quality indicators were used to measure potentially inappropriate end-of-life care. Potentially inappropriate end-of-life care was assumed to be present if one of the following six items was found in the last 30 days of life: ≥2 emergency room visits; ≥2 hospital admissions; >14 days of hospitalization; chemotherapy (in case of cancer diagnosis); ICU admission; or hospital death. These indicators, adapted from Earle et al. (2004) [10], were used because they have been used to evaluate end of life care in previous studies in the Netherlands [14,15,38].

Healthcare costs were the actual claim costs reimbursed by insurers for the last 30 days of life and included hospital care, general practitioner services, home care, and other costs such as nursing homes and expensive drugs (Supplementary file 4). The package of services in a DTC leads to one reimbursement claim that can be claimed no later than three months after the start of the first service. To include the hospital costs of only the last 30 days of life, DTC costs were divided equally over the months between the start of the first service and the date of claim. All other costs were reimbursed at the time of delivery.

### Statistical analysis

Descriptive statistics were used to describe sociodemographic characteristics, potentially inappropriate end-of-life care, and healthcare costs in the last 30 days of life (% for categorical variables and mean and median (interquartile range (IQR)) for interval variables). All deceased adults in the five regions of the initiative before the start of the implementation of the intervention were compared with all deceased adults after the start (pre-post design). As the study population included all deceased adults, including those who died unexpectedly, a sensitivity analysis was conducted by performing the same analysis on the subpopulation of deceased adults with cancer (Supplementary File 5). Moreover, deceased adults who received integrated palliative care through one of the five initiatives were compared to a 1:2 matched control group. The control group consisted of deceased adults with cancer who lived in one of the regions where the five initiatives were set but did not receive care from the initiative. An exact match was performed according to age (in 10 years categories), gender, cancer diagnosis (yes or no), year of death, and region. A chi-square test was used to test for differences in the total score and all separate items of inappropriate end-of-life care between the groups. No formal power and sample size analysis was performed, as a population-based approach comparing the regions pre and post-implementation of the intervention was used. Moreover, for the comparison between the intervention and control group, a priori, no consensus is present on what minimal difference is clinically relevant. In this study a difference of 10% was considered to be clinically relevant. All patients who received care in the initiatives were planned to be included in the analysis. This resulted in 210 patients in the intervention group and 420 in the matched control group. A sample size of 615 was needed to detect a difference of 10% with an alpha of 0.05 and power of 80%, resulting in 205 vs 410 patients using a 2:1 ratio.

Comparisons of healthcare costs in the last 30 days of life were based on average costs. To test for differences in healthcare costs, the Mann-Whitney test (Wilcoxon rank test) was used. As the distribution of healthcare costs is skewed [39], a sensitivity analysis was conducted by excluding potential outlier patients (top 1% of costs) (Supplementary File 6).

## Results

In total 37,468 adults died in the regions of the five included initiatives during the time period 2015–2019. Of these deceased adults, 17,920 died before the implementation of the intervention and 19,548 died after implementation. After implementation, of the deceased adults who received integrated care in one of the five initiatives, 210 could be matched within the database based on the information provided by the initiatives. They were matched with 420 deceased adults (Supplementary File 7).

### Characteristics of study population

Table 1 shows the sociodemographic characteristics of all deceased adults in the regions of the selected initiatives before and after the implementation of the intervention as well as of deceased adults who received integrated palliative care by one of the initiatives and the control group. After implementation, the overall majority of deceased adults were aged over 80 years (56.8%). Of the deceased adults who received integrated palliative care, 32.4% were over 80 years of age. Of all deceased adults in the regions post-implementation 29.0% had cancer as opposed to more than two-thirds (69.1%) of the deceased adults who received integrated palliative care.

**Table 1:** Sociodemographic and clinical characteristics of deceased adults in the regions of the initiatives (n = 37,468).


	DECEASED ADULTS IN THE REGIONS OF SELECTED INITIATIVES PRE-IMPLEMENTATION n = 17,920 % (n)	DECEASED ADULTS IN THE REGIONS OF SELECTED INITIATIVES POST-IMPLEMENTATION n = 19,548 % (n)	RECEIVED INTEGRATED PALLIATIVE CARE n = 210 % (n)	MATCHED CONTROL GROUP n = 420 % (n)

**Age (years)**				

18–69	20.9% (3,750)	20.4% (3,995)	35.2% (74)	35.2% (148)

70–79	21.5% (3,856)	22.7% (4,445)	32.4% (68)	32.4% (136)

80–89	35.6% (6,378)	36.6% (7,144)	25.7% (54)	25.7% (108)

90+	22.0% (3,936)	20.2% (3,964)	6.7% (14)	6.7% (28)

**Sex**				

Female	52.0% (9,319)	51.3% (10,018)	50.5% (106)	50.5% (212)

**Cancer**				

Yes	29.6% (5,295)	29.0% (5,662)	69.1% (145)	69.1% (290)

No	70.5% (12,625)	71.0% (13,886)	31.0% (65)	31.0% (130)


### Potentially inappropriate end-of-life care

**Figure 1:**
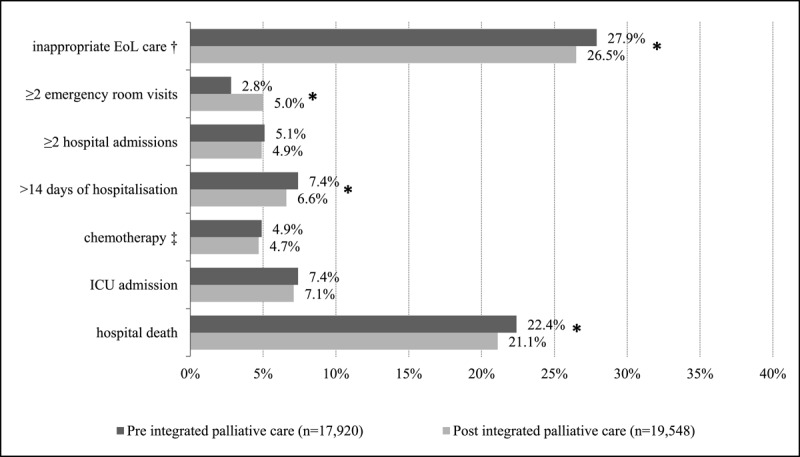
Potentially inappropriate end-of-life care of deceased adults pre-implementation (n = 17,920) compared to post-implementation (n = 19,548). All items are measured 30 days before death, except for in-hospital death. † Total score if one of the six items was obtained. ‡ Only for deceased adults with cancer. * Statistically significant difference (p < 0.05). Eol, end of life; ICU, intensive care unit.

**Figure 2:**
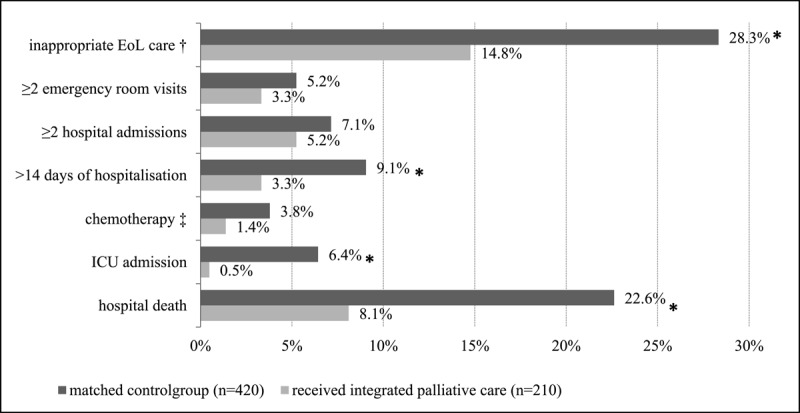
Potentially inappropriate end-of-life care of deceased adults who received integrated palliative care (n = 210) compared to a matched control group (n = 420). All items are measured 30 days before death, except for in-hospital death. † Total score if one of the six items was obtained. ‡ Only for deceased adults with cancer. * Statistically significant difference (p < 0,05). Eol, end of life; ICU, intensive care unit.

Post-implementation, significantly fewer deceased adults received inappropriate end-of-life care (26.5%) than pre-implementation (27.9%, p < 0.05). Deceased adults in the post-implementation group had more emergency room visits than those in the pre-implementation group (Figure 1). Moreover, deceased adults who received integrated palliative care (n = 210) received potentially inappropriate end-of-life care significantly less often than the matched controls (14.8% vs. 28.3%, p < 0.05). Deceased adults in the intervention group were significantly less likely to be hospitalized for more than 14 days, admitted to the ICU, and die in-hospital compared with the matched control group (Figure 2). Deceased adults with cancer who received integrated palliative care (n = 145) also received significantly less inappropriate end-of-life care (13.8%, p < 0.05) than deceased adults in the matched control group with cancer (28.6%, p < 0.05) (Supplementary File 5).

### Healthcare costs

**Figure 3:**
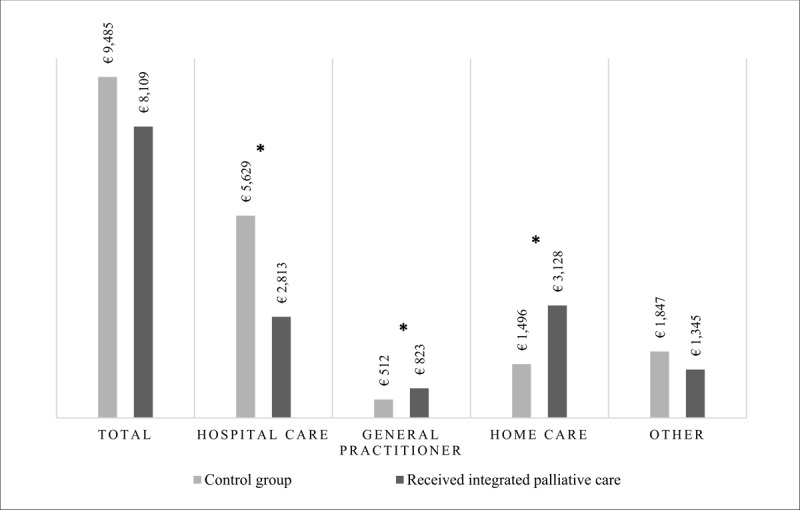
Mean healthcare costs in the last 30 days of deceased adults who received integrated palliative care (n = 210) compared to the matched control group who did not receive integrated palliative care (n = 420). * Statistically significant difference (p < 0,05).

The mean healthcare cost in the last 30 days of life were €8,109 per person (median: €6,449; IQR, €7,172) for the 210 deceased adults receiving integrated palliative care and €9,485 (median: €6,576; IQR, €7,662) for the matched control group (Figure 3). For deceased adults receiving integrated palliative care, the mean cost for hospital care was €2,813 (median €1,053; IQR, 3,694) and for home care €3,128 (median €1,844; IQR, 3,637). For the matched control group, the mean cost of hospital care was €5,629 (median: €2,057; IQR, €6,168) and for home care €1,496 (median: €98; IQR, €1,683). The differences among hospital, general practitioner, and home care were statistically significant. These differences in costs remained statistically significant when outliers in costs (top 1%) were excluded, (Supplementary File 6).

## Discussion

To our knowledge, this is the first observational study to assess the potentially inappropriate end-of-life care and healthcare costs of multiple initiatives in integrated palliative care across all care settings in various regions of the Netherlands using a population-based health insurance claims database. Integrated palliative care results in less inappropriate end-of-life care, particularly regarding hospital deaths. Moreover, the total healthcare costs of integrated palliative care are similar to those of standard care, but the costs shift from hospital to general practitioner and home care.

The results are in line with those of previous studies showing that various palliative care interventions, mostly in single care settings, are also associated with less potentially inappropriate end-of-life care [12,14,20,38,40,41]. For example, a previous Dutch study showed that adults with cancer who died in 2017 after having been provided with different forms of palliative care in different care settings had less potentially inappropriate end-of-life care in the last 30 days prior to death than deceased adults who had not (16.0% vs. 45.0%) [14]. The association between integrated palliative care and each of the separate indicators of potentially inappropriate end-of-life care was also consistent with previous research. For example, a Belgian retrospective cohort study showed that patients receiving palliative care at home died less often in the hospital than those who were not provided with palliative care at home (39.0% vs. 74.8%, respectively) [19]. In the present study, in-hospital death was low before implementation (22.4%) and in the matched control group (22.6%) compared to previous international studies, but it was even lower with integrated palliative care (21.1% and 8.1 %, respectively). One exception must be mentioned, as the percentage of ≥2 emergency room visits was significantly higher post- (5.0%) than compared to pre-implementation (2.8%), while previous studies reported a reduction of this indicator [9,10,18,41]. This unexpected increase in emergency room visits in the last month of life may be due to differences in the population (regarding diagnosis and age) [42], as the pre-post comparison was not corrected for these differences.

Several systematic literature reviews of cost and cost-effectiveness have shown that palliative care provision is associated with cost-savings [43,44,45]. This finding was confirmed by the results of the present study. The reduction of costs is highly correlated with the outcome of less potentially inappropriate end-of-life care, as this end-of-life care mainly includes hospital care and hospital care is always the highest cost category [46]. In the abovementioned study, mean healthcare costs in the last 14 days of life were lower for patients receiving palliative care at home (€3,081) than those who did not (€4,698) [19]. This difference in costs (€1,617) is consistent with that in the present study (€1,377). The present study also shows a significant decrease in mean hospital costs (€2,817) and significant increases in mean costs for general practitioner services (€311) and home care (€1,632) in the last 30 days of life. To the best of our knowledge, no other study has examined the shifts in different cost groups, except for a Canadian study of 107,253 patients who died of cancer between 2005 and 2009 [11]. This Canadian study compared different cost categories in the last month of life between patients who received potentially inappropriate end-of-life care and those who did not and found a shift from mean hospital costs (€4,570) to home care (€382). The shift in costs could be explained by the treatment and care for patients receiving integrated palliative care being less focused on treating the disease and more on quality of life and therefore requiring less treatment in the hospital and more care at home.

The integration of all services involved in a patient care trajectory is considered challenging as its key features are extensive [33] and its development is time consuming and complex [47]. However, the findings of this study support the need to overcome these challenges, as integrated palliative care seems to have the potential to further reduce potentially inappropriate end-of-life care without increasing healthcare costs. Further evidence is needed regarding which elements of integrated palliative care, as a complex intervention, are most effective. As there was a high service variation across the five initiatives, assessing the impact on potentially inappropriate end-of-life care and healthcare costs of each individual initiative might be useful to gain insight into the effectiveness of each initiative compared to usual care and which model can be considered the best practice. It may also be helpful to gain insight into suitable implementation strategies and the impact of the degree of maturity of these integrated palliative care initiatives on its effectiveness. Furthermore, potentially inappropriate end-of-life care is an indicator of a lower quality of care. More insights are needed in other outcomes such as patient-reported outcomes and appropriate end-of-life care to further validate the results of this study. This study was conducted before the COVID-19 pandemic. However, other research has shown that fewer patients received potentially inappropriate end-of-life care during the pandemic [48]. It would be interesting to assess to what extent this reduction of potentially inappropriate end-of-life care is related to more integrated palliative care provision.

The shift in costs from hospital care to general practitioner services and home care may indicate a shift in care from the former to the latter in line with patients’ preferences for dying at home [49]. To further encourage this shift, the health system needs to be organized accordingly, for example, by developing suitable payment reforms. Moreover, further research on the effects of integrated palliative care in a larger and more representative sample is needed to substantiate the results of this study.

### Limitations

Some limitations of this study should be considered when interpreting the results. First, owing to the use of nationwide health insurance claims data, limited clinical information was available. Therefore, all deceased adults were included, not only patients suffering from non-communicable diseases or frailty and therefore potentially in need of palliative care, but also adults who died unexpectedly. In high-income countries, 69%–82% of deceased people are potentially in need of palliative care [50]. This may have led to an over- or underestimation of our results, depending on the distribution of adults who died unexpectedly among the compared groups. Second, because of limited clinical information, only a number patients and disease characteristics were available to match with the control group. The claims data did not include ICD10 codes and apart from cancer, the illness of patients could not adequately be derived from the diagnoses codes within the DTC’s as these codes can cover multiple illnesses. For example, dementia is classified by a code that is also used to classify memory problems. Therefore, residual confounding factors are likely to be present. Third, the Dutch government has included national palliative care programs in its health policy to improve awareness of palliative care needs [51]. This may have contributed to the differences in potentially inappropriate end-of-life care between the pre- and post-implementation periods. Fourth, the number of deceased adults who received integrated palliative care was rather small due to limited referrals to integrated palliative care and the failure to identify some patients in the Vektis database. Moreover, selection bias was present in the intervention group, as patients receiving integrated palliative care were younger and had cancer more often than the entire patient group [52]. This hampers the generalizability of the results.

Considering healthcare costs, as there is currently no clear reimbursement scheme for integrated palliative care in the Netherlands [53], intervention costs may not all be present in the claims database. This may have led to an underestimation of healthcare costs in the last month of life for patients receiving integrated palliative care. Our analysis included only healthcare costs; the financial value of care provided by the family and unpaid caregivers (informal care costs) was not included in the cost analysis. Therefore, to fully understand the cost-effectiveness of integrated palliative care, future research should consider its impact on broader societal costs. Furthermore, it was not possible to correct for price differences among providers based in the available data. In the Netherlands, prices are negotiated, in part, between providers and health insurers and are therefore different for each provider. This could lead to both underestimation and overestimation of mean healthcare costs. However, this may only have a limited effect on the outcome, as the reduction in costs is mainly due to the lower costs of hospital care, which is always the highest cost category. Finally, as DTC’s were divided equally over the months between opening and the moment they were claimed, this may have led to an underestimation of hospital costs in the last month of life, as these costs were shown to be higher in the last month of life than in the previous months [46].

## Conclusion

This study shows less potentially inappropriate end-of-life care and a shift in healthcare costs in the last month of life from hospital to general practitioner and home care in patients receiving integrated palliative care. These results highlight the importance of implementation of integrated palliative care into standard care and the development of suitable payment reforms. Further research on the effects of integrated palliative care in a larger and more representative sample is necessary to substantiate the results of this study.

## Additional File

The additional file for this article can be found as follows:

10.5334/ijic.7504.s1 Supplementary Files. Supplementary Files 1 to 7.

## References

[1] Sleeman KE, de Brito M, Etkind S, Nkhoma K, Guo P, Higginson IJ, et al. The escalating global burden of serious health-related suffering: projections to 2060 by world regions, age groups, and health conditions. Lancet Glob Health. 2019; 7(7): e883–e92. DOI: 10.1016/S2214-109X(19)30172-X 31129125 PMC6560023

[2] Sepulveda C, Marlin A, Yoshida T, Ullrich A. Palliative Care: the World Health Organization’s global perspective. J Pain Symptom Manage. 2002; 24(2): 91–6. DOI: 10.1016/S0885-3924(02)00440-2 12231124

[3] Huo B, Song Y, Chang L, Tan B. Effects of early palliative care on patients with incurable cancer: A meta-analysis and systematic review. Eur J Cancer Care (Engl). 2022; 31(6): e13620. DOI: 10.1111/ecc.13620 35612356

[4] Hoomani Majdabadi F, Ashktorab T, Ilkhani M. Impact of palliative care on quality of life in advanced cancer: A meta-analysis of randomised controlled trials. Eur J Cancer Care (Engl). 2022; 31(6): e13647. DOI: 10.1111/ecc.13647 35830961

[5] Temel JS, Greer JA, Muzikansky A, Gallagher ER, Admane S, Jackson VA, et al. Early palliative care for patients with metastatic non-small-cell lung cancer. N Engl J Med. 2010; 363(8): 733–42. DOI: 10.1056/NEJMoa1000678 20818875

[6] Bajwah S, Oluyase AO, Yi D, Gao W, Evans CJ, Grande G, et al. The effectiveness and cost-effectiveness of hospital-based specialist palliative care for adults with advanced illness and their caregivers. Cochrane Database Syst Rev. 2020; 9: CD012780. DOI: 10.1002/14651858.CD012780.pub2 32996586 PMC8428758

[7] Vanbutsele G, Van Belle S, Surmont V, De Laat M, Colman R, Eecloo K, et al. The effect of early and systematic integration of palliative care in oncology on quality of life and health care use near the end of life: A randomised controlled trial. Eur J Cancer. 2020; 124: 186–93. DOI: 10.1016/j.ejca.2019.11.009 31812934

[8] Huang YT, Wang YW, Chi CW, Hu WY, Lin R, Jr., Shiao CC, et al. Differences in medical costs for end-of-life patients receiving traditional care and those receiving hospice care: A retrospective study. PLoS One. 2020; 15(2): e0229176. DOI: 10.1371/journal.pone.0229176 32078660 PMC7032706

[9] Maetens A, Beernaert K, De Schreye R, Faes K, Annemans L, Pardon K, et al. Impact of palliative home care support on the quality and costs of care at the end of life: a population-level matched cohort study. BMJ Open. 2019; 9(1): e025180. DOI: 10.1136/bmjopen-2018-025180 PMC6347879 30670524

[10] Scibetta C, Kerr K, McGuire J, Rabow MW. The Costs of Waiting: Implications of the Timing of Palliative Care Consultation among a Cohort of Decedents at a Comprehensive Cancer Center. J Palliat Med. 2016; 19(1): 69–75. DOI: 10.1089/jpm.2015.0119 26618636

[11] Cheung MC, Earle CC, Rangrej J, Ho TH, Liu N, Barbera L, et al. Impact of aggressive management and palliative care on cancer costs in the final month of life. Cancer. 2015; 121(18): 3307–15. DOI: 10.1002/cncr.29485 26031241 PMC4560956

[12] Earle CC, Neville BA, Landrum MB, Ayanian JZ, Block SD, Weeks JC. Trends in the aggressiveness of cancer care near the end of life. J Clin Oncol. 2004; 22(2): 315–21. DOI: 10.1200/JCO.2004.08.136 14722041

[13] Qureshi D, Tanuseputro P, Perez R, Pond GR, Seow HY. Early initiation of palliative care is associated with reduced late-life acute-hospital use: A population-based retrospective cohort study. Palliat Med. 2019; 33(2): 150–9. DOI: 10.1177/0269216318815794 30501459 PMC6399729

[14] Boddaert MS, Pereira C, Adema J, Vissers KCP, van der Linden YM, Raijmakers NJH, et al. Inappropriate end-of-life cancer care in a generalist and specialist palliative care model: a nationwide retrospective population-based observational study. BMJ Support Palliat Care; 2020. DOI: 10.1136/bmjspcare-2020-002302 PMC9120402 33355176

[15] De Schreye R, Houttekier D, Deliens L, Cohen J. Developing indicators of appropriate and inappropriate end-of-life care in people with Alzheimer’s disease, cancer or chronic obstructive pulmonary disease for population-level administrative databases: A RAND/UCLA appropriateness study. Palliat Med. 2017; 31(10): 932–45. DOI: 10.1177/0269216317705099 28429629

[16] Zhang B, Nilsson ME, Prigerson HG. Factors important to patients’ quality of life at the end of life. Arch Intern Med. 2012; 172(15): 1133–42. DOI: 10.1001/archinternmed.2012.2364 22777380 PMC3806298

[17] Shrank WH, Rogstad TL, Parekh N. Waste in the US Health Care System: Estimated Costs and Potential for Savings. JAMA. 2019; 322(15): 1501–9. DOI: 10.1001/jama.2019.13978 31589283

[18] Hui D, Kim SH, Roquemore J, Dev R, Chisholm G, Bruera E. Impact of timing and setting of palliative care referral on quality of end-of-life care in cancer patients. Cancer. 2014; 120(11): 1743–9. DOI: 10.1002/cncr.28628 24967463 PMC4073257

[19] Maetens A, Deliens L, De Bleecker J, Caraceni A, De Ridder M, Beernaert K, et al. Healthcare utilization at the end of life in people dying from amyotrophic lateral sclerosis: A retrospective cohort study using linked administrative data. J Neurol Sci. 2019; 406: 116444. DOI: 10.1016/j.jns.2019.116444 31520967

[20] De Schreye R, Smets T, Deliens L, Annemans L, Gielen B, Cohen J. Appropriateness of End-of-Life Care in People Dying With Dementia: Applying Quality Indicators on Linked Administrative Databases. J Am Med Dir Assoc. 2020; 21(8): 1093–101 e1. DOI: 10.1016/j.jamda.2019.12.020 32037298

[21] Monitor palliative care. Dutch Healthcare Authority; 2020.

[22] Boddaert MS, Douma J, Dijxhoorn AQ, Heman R, van der Rijt CCD, Teunissen S, et al. Development of a national quality framework for palliative care in a mixed generalist and specialist care model: A whole-sector approach and a modified Delphi technique. PLoS One. 2022; 17(3): e0265726. DOI: 10.1371/journal.pone.0265726 35320315 PMC8942240

[23] van Riet Paap J, Vernooij-Dassen M, Brouwer F, Meiland F, Iliffe S, Davies N, et al. Improving the organization of palliative care: identification of barriers and facilitators in five European countries. Implement Sci. 2014; 9: 130. DOI: 10.1186/s13012-014-0130-z 25686479 PMC4203898

[24] van Roij J, Raijmakers N, Ham L, van den Beuken-van Everdingen M, van den Borne B, Creemers GJ, et al. Quality of life and quality of care as experienced by patients with advanced cancer and their relatives: A multicentre observational cohort study (eQuiPe). Eur J Cancer. 2022; 165: 125–35. DOI: 10.1016/j.ejca.2022.01.039 35235869

[25] Stoop A, Lette M, Ambugo EA, Gadsby EW, Goodwin N, MacInnes J, et al. Improving Person-Centredness in Integrated Care for Older People: Experiences from Thirteen Integrated Care Sites in Europe. Int J Integr Care. 2020; 20(2): 16. DOI: 10.5334/ijic.5427 PMC7319083 32607103

[26] Kodner DL. All together now: a conceptual exploration of integrated care. Healthc Q. 2009; 13 Spec No: 6–15. DOI: 10.12927/hcq.2009.21091 20057243

[27] Bloem BR, Munneke M. Revolutionising management of chronic disease: the ParkinsonNet approach. BMJ. 2014; 348: g1838. DOI: 10.1136/bmj.g1838 24647365

[28] Stelwagen MA, van Kempen A, Westmaas A, Blees YJ, Scheele F. Integration of Maternity and Neonatal Care to Empower Parents. J Obstet Gynecol Neonatal Nurs. 2020; 49(1): 65–77. DOI: 10.1016/j.jogn.2019.11.003 31809695

[29] World Health Organization. Integrated care models: an overview. Working document; 2016.

[30] Pereira CFR, de Wit A, Middelburg M, Boddaert MS, Raijmakers NJH. Identification and assessment of integrated palliative care initiatives in the Netherlands. 16th World congress of the European association for palliative care; Berlin; 2019. DOI: 10.26226/morressier.5c76c8bde2ea5a723761287b

[31] Engel M, Stoppelenburg A, van der Ark A, Bols FM, Bruggeman J, Janssens-van Vliet ECJ, et al. Development and implementation of a transmural palliative care consultation service: a multiple case study in the Netherlands. BMC Palliat Care. 2021; 20(1): 81. DOI: 10.1186/s12904-021-00767-6 34090394 PMC8180007

[32] van Doorne I, van Schie VMW, Parlevliet JL, Willems DL, van Rijn M, Buurman BM. Challenges in the implementation and evaluation of a transmural palliative care pathway for acutely hospitalized older adults; lessons from the PalliSupport program: A qualitative process evaluation study. Arch Gerontol Geriatr. 2022; 103: 104782. DOI: 10.1016/j.archger.2022.104782 35917717

[33] Valentijn PP, Boesveld IC, van der Klauw DM, Ruwaard D, Struijs JN, Molema JJ, et al. Towards a taxonomy for integrated care: a mixed-methods study. Int J Integr Care. 2015; 15: e003. DOI: 10.5334/ijic.1513 25759607 PMC4353214

[34] IKNL/Palliactief. Netherlands Quality Framework for Palliative Care; 2017.

[35] Quill TE, Abernethy AP. Generalist plus specialist palliative care--creating a more sustainable model. N Engl J Med. 2013; 368(13): 1173–5. DOI: 10.1056/NEJMp1215620 23465068

[36] Boddaert MS, Stoppelenburg A, Hasselaar J, van der Linden YM, Vissers KCP, Raijmakers NJH, et al. Specialist palliative care teams and characteristics related to referral rate: a national cross-sectional survey among hospitals in the Netherlands. BMC Palliat Care. 2021; 20(1): 175. DOI: 10.1186/s12904-021-00875-3 34758792 PMC8582112

[37] World Health Organization. Continuity and coordination of care; 2018.

[38] Bolt EE, Pasman HR, Willems D, Onwuteaka-Philipsen BD. Appropriate and inappropriate care in the last phase of life: an explorative study among patients and relatives. BMC Health Serv Res. 2016; 16(1): 655. DOI: 10.1186/s12913-016-1879-3 27846832 PMC5111254

[39] Mihaylova B, Briggs A, O’Hagan A, Thompson SG. Review of statistical methods for analysing healthcare resources and costs. Health Econ. 2011; 20(8): 897–916. DOI: 10.1002/hec.1653 20799344 PMC3470917

[40] De Schreye R, Smets T, Deliens L, Annemans L, Gielen B, Cohen J. Appropriateness of End-of-Life Care in People Dying From COPD. Applying Quality Indicators on Linked Administrative Databases. J Pain Symptom Manage. 2018; 56(4): 541–50 e6. DOI: 10.1016/j.jpainsymman.2018.06.011 29960021

[41] Ho TH, Barbera L, Saskin R, Lu H, Neville BA, Earle CC. Trends in the aggressiveness of end-of-life cancer care in the universal health care system of Ontario, Canada. J Clin Oncol. 2011; 29(12): 1587–91. DOI: 10.1200/JCO.2010.31.9897 21402603 PMC3082976

[42] Oosterveld, MAR, Heins M, Boddaert M, Engels Y, van der Heide A, Onwuteaka-Philipsen B, Verheij R, Francke A. Palliative care in the Netherlands: Facts and figures. Factsheet 2: Acute care in the hospital and the general practice; 2020.

[43] Smith S, Brick A, O’Hara S, Normand C. Evidence on the cost and cost-effectiveness of palliative care: a literature review. Palliat Med. 2014; 28(2): 130–50. DOI: 10.1177/0269216313493466 23838378

[44] Langton JM, Blanch B, Drew AK, Haas M, Ingham JM, Pearson SA. Retrospective studies of end-of-life resource utilization and costs in cancer care using health administrative data: a systematic review. Palliat Med. 2014; 28(10): 1167–96. DOI: 10.1177/0269216314533813 24866758

[45] Mathew C, Hsu AT, Prentice M, Lawlor P, Kyeremanteng K, Tanuseputro P, et al. Economic evaluations of palliative care models: A systematic review. Palliat Med. 2020; 34(1): 69–82. DOI: 10.1177/0269216319875906 31854213

[46] Alipour V, Pourreza A, Kosheshi M, Heydari H, Emamgholipour Sefiddashti S. Hospital Expenditure at the End-of-Life: A Time-to-Death Approach. Int J Health Policy Manag. 2022; 11(2): 138–44. 32610810 10.34172/ijhpm.2020.88 PMC9278609

[47] van Vooren NJE, Steenkamer BM, Baan CA, Drewes HW. Transforming towards sustainable health and wellbeing systems: Eight guiding principles based on the experiences of nine Dutch Population Health Management initiatives. Health Policy. 2020; 124(1): 37–43. DOI: 10.1016/j.healthpol.2019.11.003 31806356

[48] Slotman E, Fransen HP, van Laarhoven HW, van den Beuken-van Everdingen MH, Tjan-Heijnen VC, Huijben AM, et al. Reduction in potentially inappropriate end-of-life hospital care for cancer patients during the COVID-19 pandemic: A retrospective population-based study. Palliat Med. 2024; 38(1): 140–9. DOI: 10.1177/02692163231217373 38142283 PMC10798006

[49] The Choice in End of Life Care Programme Board. What’s important to me. A review of choice in end of lifecare. 2015.

[50] Murtagh FE, Bausewein C, Verne J, Groeneveld EI, Kaloki YE, Higginson IJ. How many people need palliative care? A study developing and comparing methods for population-based estimates. Palliat Med. 2014; 28(1): 49–58. DOI: 10.1177/0269216313489367 23695827

[51] I heard I will die, but untill then, I am alive [Available from: https://overpalliatievezorg.nl/].

[52] Etkind SN, Bone AE, Gomes B, Lovell N, Evans CJ, Higginson IJ, et al. How many people will need palliative care in 2040? Past trends, future projections and implications for services. BMC Med. 2017; 15(1): 102. DOI: 10.1186/s12916-017-0860-2 28514961 PMC5436458

[53] Coöperation in palliative care. Care around the patient: Dutch Healthcare Authority; 2018.

