# Supplementary material for: Potentially Inappropriate End of Life Care and Healthcare Costs in the Last 30 Days of Life in Regions Providing Integrated Palliative Care in the Netherlands: A Registration-based Study
Source: Int J Integr Care. 2024 Jul 8;24(3):6. doi: 10.5334/ijic.7504 (PMC11243768; doi:10.5334/ijic.7504)

Supplementary File 1

Selection of Integrated Palliative Care initiatives in the Netherlands

Method:

- Survey among all palliative care networks in the Netherlands and regional advisors of Fibula Foundation (currently named Netherlands Association for Palliative Care (PZNL)
- Inquiry of documents of all identified initiatives
- Assessment of these documents to assess the level of implementation of 8 key elements of the Netherlands Quality framework for Palliative Care
- Assessment by two independent researchers
- Consensus meeting

Assessment form:

1	**Identification of palliative care needs**<br><br>In the palliative phase, quality of life and dying are paramount and the pros and cons of treatment may be perceived differently. Such a change of treatment goal should be identified and discussed with the patient.	0. No arrangements are present on identification<br>1. Arrangements have been recorded on identification<br>2. Arrangements have been recorded about identification using an instrument<br>3. Arrangements have been recorded about registering the identification in the patient file
2	**Shared decision-making**<br><br>Shared decision-making is the process in which the healthcare provider and the patient jointly discuss which care suits the patient best, taking into account all options, pros and cons, preferences and circumstances of the patient.	0. No arrangements have been established on shared decision-making<br>1. Arrangements have been recorded on shared decision-making<br>2. Arrangements have been recorded on repeated tuning based on shared decision-making<br>3. Arrangements have been recorded on how to record the outcomes of shared decision-making in the file.
3	**Advance care planning**<br><br>Advance care planning is the process of thinking ahead, planning and organising.	0. No arrangements have been established on advance care planning<br>1. Arrangements have been recorded on advance care planning<br>2. Arrangements have been recorded on repeated coordination about the process of advance care planning<br>3. Arrangements are present on how to record the outcomes of the advance care planning process in the patient file.
4	**Coordination and continuity of care**	

	The central care provider is the main point of contact for the patient and their relatives and the entire team of care providers involved.	0. No arrangements have been made for a central care provider<br>1. Arrangements have been recorded about a central care provider<br>2. Arrangements have been recorded regarding the responsibilities and tasks of the central care provider<br>3. Arrangements have been recorded about who the central care provider is and this is clear to all involved (patient, relatives and healthcare professionals).
5	**Individual care plan**<br><br>The individual care plan is the dynamic set of agreements between the patient and the healthcare provider(s) on care and self-management.	0. There is no individual care plan<br>1. Arrangements on the use of an individual care plan have been recorded<br>2. Arrangements have been recorded regarding the accessibility of the individual care plan for patients, relatives and all healthcare professionals involved<br>3. Arrangements are recorded regarding the digital availability of the individual care plan for patient, relatives and all involved healthcare professionals.
6	**Expertise**<br><br>Every caregiver involved in providing palliative care, at generalist, specialist or expert level, therefore follows appropriate continuing education and training to ensure quality of palliative care.	0. No arrangements have been recorded regarding the expertise of the healthcare professionals involved<br>1. Arrangements have been recorded regarding the expertise of the involved healthcare professionals<br>2. Arrangements have been recorded regarding the deployment of healthcare professionals specialised in palliative care<br>3. Arrangements are recorded regarding structural collaboration between generalist and specialist palliative care professionals (for example PaTz or a shared MCM).
7	**(Effective) communication**<br><br>Effective communication involves a structured process between the patient and the healthcare provider, in which two-way information is exchanged and equality - with respect for the dependent position of the patient - is the basis.	0. No attention has been paid to (effective) communication<br>1. Attention was paid to (effective) communication
8	**Work-life balance of healthcare professionals**<br><br>Caregivers and volunteers are aware of the emotional impact that providing palliative care can have on themselves. They reflect on their own attitudes and actions and are mindful of their personal balance. In this, they take care of themselves and their colleagues.	0. There is no attention for the emotional impact on the healthcare professional of providing palliative care<br>1. Attention for the emotional impact on the healthcare professional of providing palliative care is present<br>2. There is attention for the emotional impact on the

		healthcare professional of providing palliative care and tools/interventions are used (e.g. moral reflection, peer review)<br>3. There is attention for the emotional impact on the healthcare professional for providing palliative care and the organisation has a facilitating role.
	**Total score**	0 (minimal) – 23 (maximum)

Supplementary File 2

Sociodemographic and clinical characteristics of deceased adults in the regions of the initiatives and characteristics of the initiatives during the period 2015-2019

	**1**	**2**	**3**	**4**	**5**
	n=8,335 % (n)	n=8,440 % (n)	n=2,398 % (n)	n=7,527 % (n)	n=10,768 % (n)
Region	West	Southeast	East	East	South
**Elements of integrated palliative care**					
Integrated case management			x	x	
Integrated care pathway		x		x	x
Integrated consultation team	x	x			x
**Providers involved**					
Hospital	x	x	x	x	x
General practitioner		x			x
Home care			x	x	x
Other		x†			x‡
Start of implementation	Oktober 2018	January 2016	June 2019	January 2018	January 2016
Number of months post implementation	12	45	3	21	45
**Age**					
18-69	19.66% (1,639)	20.94% (1,767)	20.56% (493)	21.02% (1,582)	21.03% (2,264)
70-79	20.79% (1,733)	21.90% (1,848)	22.35% (536)	22.43% (1,688)	23.18% (2,496)
80-89	35.27% (2,940)	37.31% (3,149)	34.70% (832)	35.15% (2,646)	36.73% (3,955)
90+	24.27% (2,023)	19.86% (1,676)	22.39% (537)	21.40% (1,611)	19.07% (2,053)
**Gender**					
Female	52.95% (4,413)	51.50% (4,347)	50.25% (1,205)	52.25% (3,933)	50.51% (5,439)
**Cancer**					
Yes	27.13% (2,261)	26.64% (2,248)	32.86% (788)	31.57% (2376)	30.50% (3,284)
No	72.87% (6,074)	73.36% (6,192)	67.14% (1,610)	68.43% (5,151)	69.50% (7,484)

† pharmacist

‡ nursing home

Supplementary File 3

Hospital care in the Netherlands is reimbursed per Diagnosis treatment combination (DTC). A DTC contains all per patient hospital services related to a diagnosis, treatment and follow-up. Cancer diagnosis was determined by the presence of a DTC related to solid tumours in the year preceding death.

An example of a cancer types and their associated DTC:

Oncology identification	***Specialist code***	***Diagnostic code***	***Oncology identification***	***Specialist code***	***Diagnostic code***	***Oncology identification***	***Specialist code***	***Diagnostic code***

Oncology identification	***Specialist code***	***Diagnostic code***	***Oncology identification***	***Specialist code***	***Diagnostic code***	***Oncology identification***	***Specialist code***	***Diagnostic code***
Cancer of breast	0303	0318	***Cancer of breast, secondary***	0304	0221	***Cancer of other respiratory & intrathoracic***	0322	1305
	0313	0811		0304	0222		0322	1306
	0361	0105		0304	0223		0322	1307
				0304	0224		0303	0314
				0304	0225		0313	0623
				0304	0226		0313	0624
				0304	0230		0313	0629

Full table is available at request from the corresponding author and is included in the supplementary files of Boddaert et al (2020).

Supplementary File 4

Different cost categories from primary and secondary care settings were included in the study:

Hospital care	
General practioner	
Home care (nursing & personal care)*	
Other, included:	Paramedical care
	Physical therapy
	Drugs
	Expensive drugs
	Short term care*
	Long term care*
	Mental care

* In the Netherlands, there is no specific reimbursement for hospice care. Expenses for home care and short and long term care also cover hospice care.

Supplementary File 5

Potentially inappropriate end of life care of deceased adults with cancer who received integrated palliative care (n=145) compared to a matched control group (n=290)

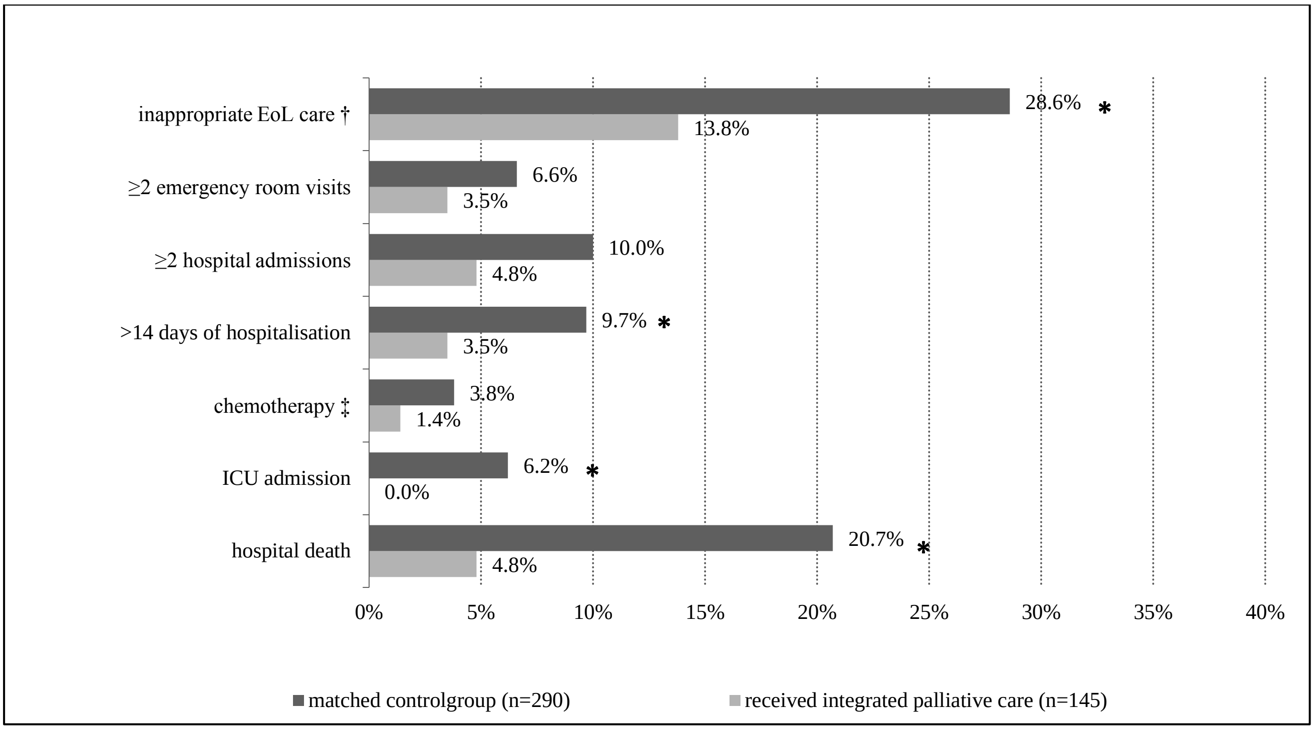

All items are measured 30 days before death, except for in-hospital death

† Total score if one of the six items was found

‡ Only for deceased adults with cancer

* Statistically significant difference (p<0,05)

Supplementary File 6

Sensitivity analysis healthcare costs: exclusion of patients with top 1 costs

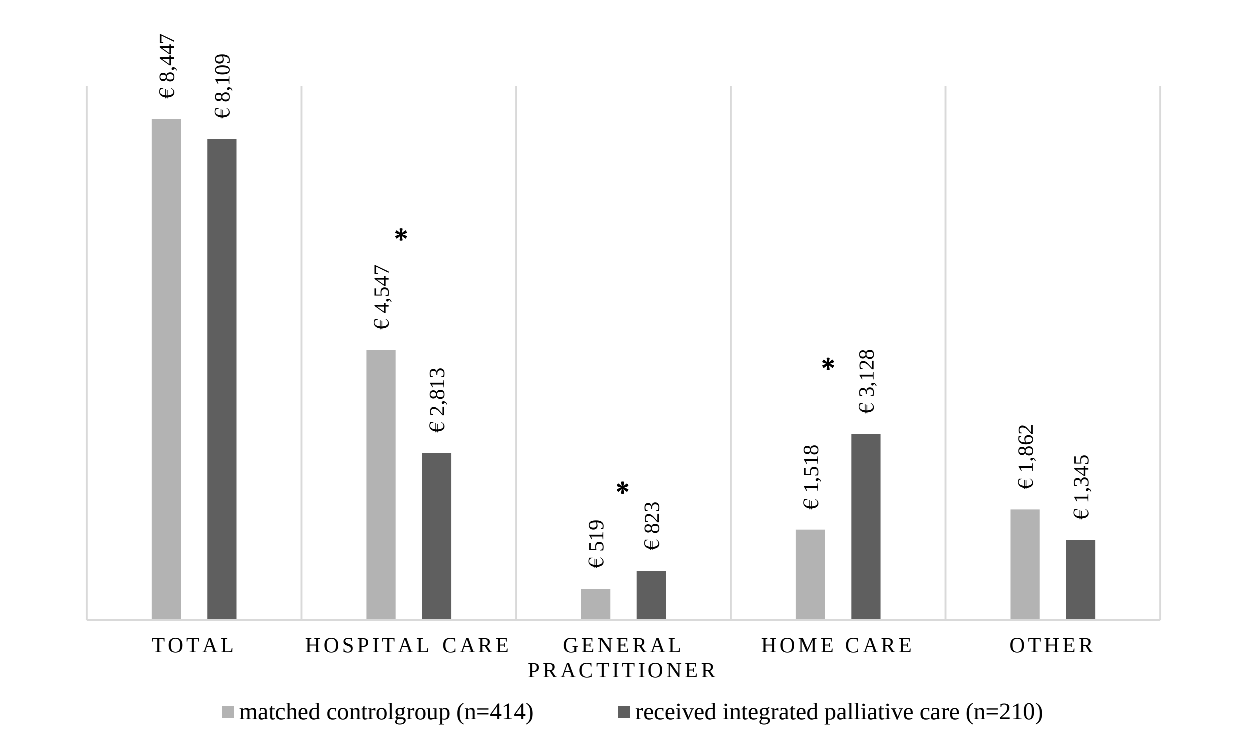

* Statistically significant difference (p<0,05)

Supplementary File 7

Flowchart study population

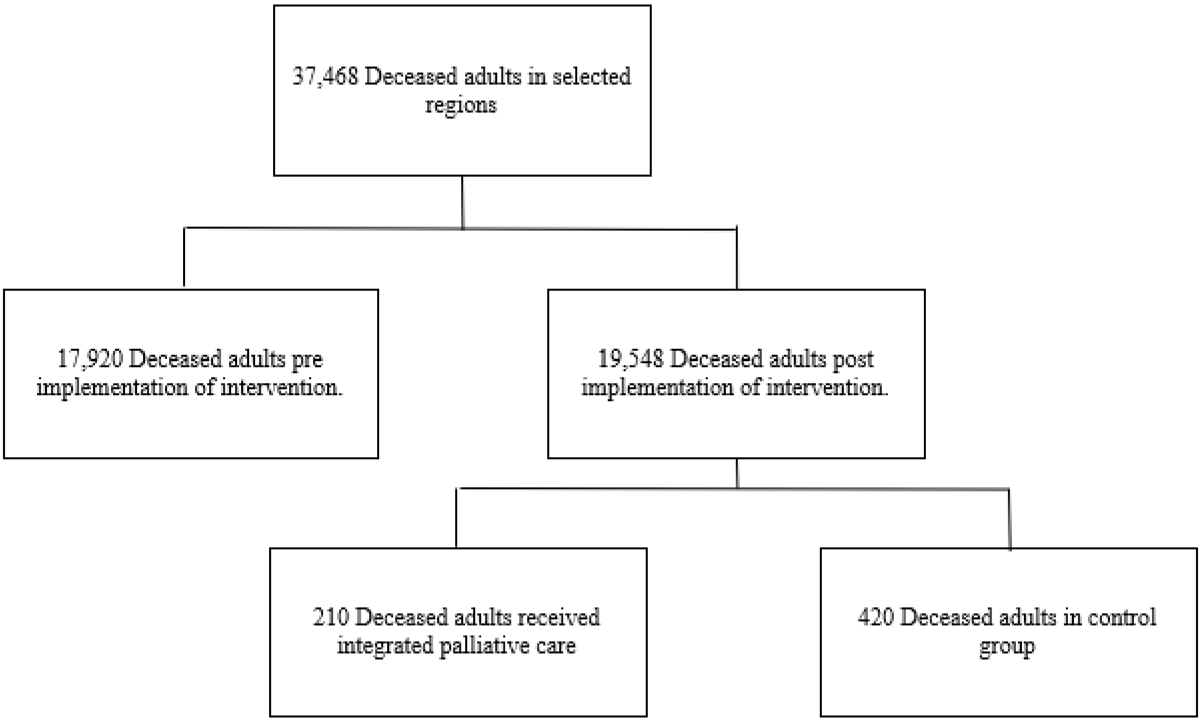

Supplement: Supplementary Files. — Supplementary Files 1 to 7. [file ijic-24-3-7504-s1.pdf]
